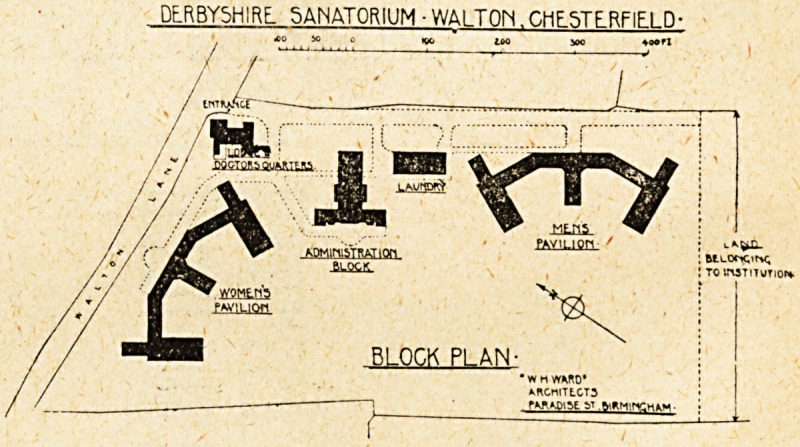# Derbyshire Sanatorium, Walton, Chesterfield

**Published:** 1916-11-04

**Authors:** 


### DERBYSHIRE SANATORIUM, WALTON, CHESTERFIELD.

This sanatorium is planned on somewhat novel lines. It comprises five separate buildings placed near the N.E. and N.W. boundaries of the site, which is about 13½ acres in extent.

The blocks comprise two for patients, an administration building, lodge and doctors' quarters, and the laundry.

The two blocks for patients are with slight variations identical, one being for male, the other for female patients. They are one storey in height, and may be described as, roughly, in the form of a capital "E," with the upright stroke bent above and below the centre

The total number of beds for patients is 100, which gives approximately a proportion of 7.4 patients per acre.

stroke at an obtuse angle inwards. The centre of the block is occupied by the duty room, on one side of which is a drying room for clothes and boots, on the other an examining room with dispensary attached. Next to the drying room is the entrance, and adjoining is a store room. On the other side of the dispensary is another drying room for clothes and boots. The remainder of the space to where the bend occurs is taken up on each side with two single-bed wards. These wards are intended for patients on admission, and are provided with hot-water radiators for heating in winter. The irregular spaces formed by the bent angles are utilised as linen stores.

From the bends to the wings which represent the top nurses, the matron's quarters, and the kitchen offices and stores. A staircase in the front of the block gives access to the nurses' bedrooms, and one at the back to the servants' bedrooms.

The lodge and doctors' quarters comprise a self-contained dwelling for the caretaker, and accommodation for doctor and assistant doctor.

The laundry is planned on the usual lines of receiving room, washhouse, drying and ironing room, and delivery room; there is also a disinfecting apparatus planned in the usual way, with separate rooms for infected and disinfected goods, and a separate receiving room. The boiler house and a small mortuary complete the accommodation in this block.

and bottom strokes of the "E" are two groups of four wards, each for two beds. These wards are not provided with any means of warming, and have less complete protection from wind than the single-bed wards.

Patients are drafted from the single-bed wards to the double-bed wards, and thence to the open-air wards, which occupy the top and bottom wings.

The centre stroke of the "E" contains the dining room, adjoining which is a pantry, china and washing-up room, serving room, and nurses' w.c. The dining room has its floor slightly warmed by means of hot-water pipes running in a closed channel under the concrete surface.

The two wings each contain a ward open to the air on two sides, and divided into two parts longitudinally by a spinal wall. A lantern-light the whole length of the ward provides light and ventilation for both sides, the spinal wall not being carried up the full height, an arrangement clearly shown by section E, F. On one side of the partition are eight beds; on the other seven beds. The rest of the wing is occupied by a large dressing room, fitted with lockers and lavatory basins, two w.c.'s, and a urinal in the men's wing, and three w.c.s in the women's wing, a sink room, an ordinary bathroom and a spray bathroom, and a side entrance.

The whole of each building is constructed on a reinforced concrete raft, with breeze concrete partition blocks, plastered inside and cemented outside.

The dining room has a wooden roof covered with slates, the remainder of the roofs being flat and covered with vulcanite.

The administration block contains on the ground floor an $x$-ray and dark room, dining and sitting room for

The site plan shows the part of the land at present occupied by buildings. In addition to this, there is about another 5½ acres available for future extension; this additional land has an exercise path running round the boundaries.

The total cost, including land, furnishing, and equipment, was nearly £22,000, and the cost of building only was £120 per bed.

The architects were Messrs. W. H. Ward, of Birmingham.

The beds in the open-air pavilion are provided with a waterproof canopy over the foot end of the bed; this cover can be erected in half a minute, and not only prevents the bed-clothes from becoming wet, but keeps the patient warm and dry. This "Barwise" sanatorium bed is made by the Smith and Cartwright Bedstead Company, Ltd., Birmingham.

## Figures and Tables

**Figure f1:**
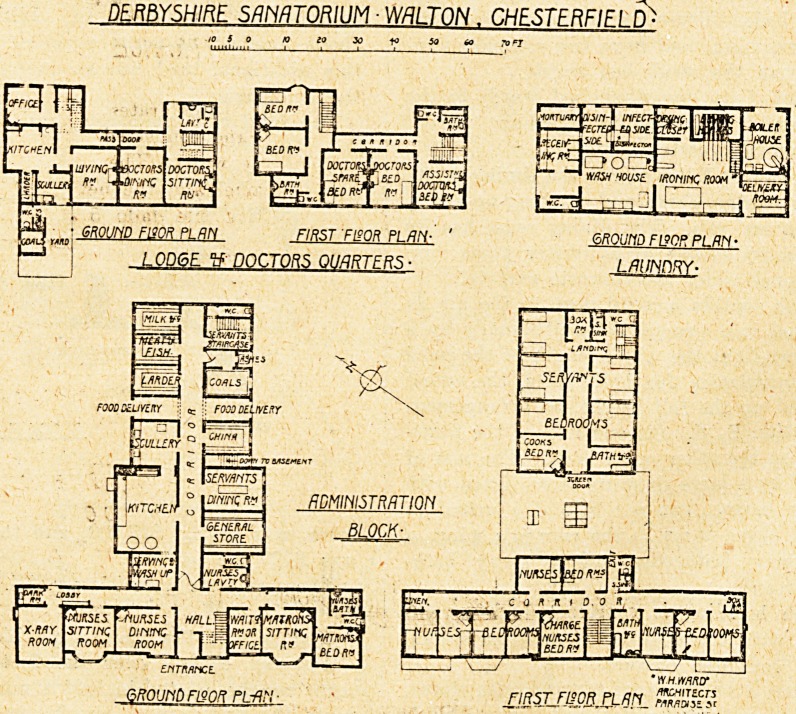


**Figure f2:**
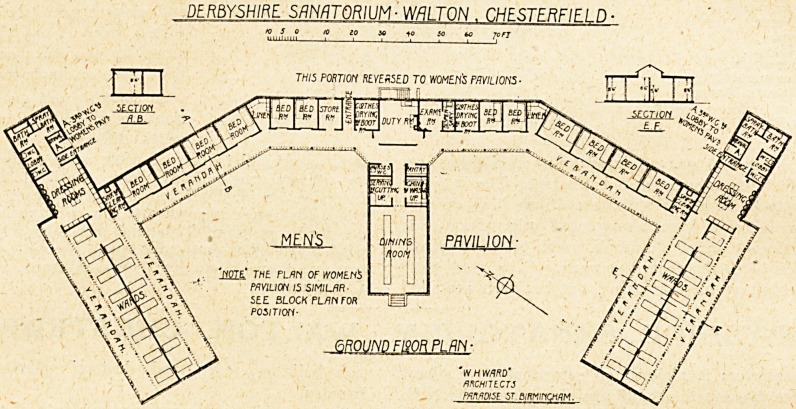


**Figure f3:**